# Predictors of mortality in acute pancreatitis complicated with multidrug-resistant *Klebsiella pneumoniae* infection

**DOI:** 10.1186/s12879-021-06709-0

**Published:** 2021-09-20

**Authors:** Di Wu, Junjie Ding, Yan Jia, Huanmiao Liu, Jie Xiao, Jie Peng

**Affiliations:** 1 Department of Gastroenterology, Xiangya Hospital, Central South University, Xiangya Road, Changsha, 410008 China; 2 Emergency Department, Third Xiangya Hospital, Central South University, Changsha, China

**Keywords:** Acute pancreatitis, Multidrug-resistant infection, Mortality, Drug-resistance, Predictor

## Abstract

**Background:**

Multidrug-resistant (MDR) *Klebsiella pneumoniae* infections, from pancreatic infections to bloodstream infections, influence the mortality of patients with acute pancreatitis (AP) on the condition of limited antibiotic choices. The aim of this study was to investigate the predictor of mortality among AP patients complicated with MDR-*K. pneumoniae* infections.

**Methods:**

Seventy-one AP patients who occurred MDR-*K. pneumoniae* infections from August 1st, 2016 to August 1st, 2020 were enrolled. MDR-*K. pneumoniae* was defined as the *K. pneumoniae* strain non-susceptible to at least one agent in three or more antimicrobial categories. MDR-*K. pneumoniae* isolates were confirmed by Vitek-2 system. Antibiotic susceptibility test was carried out using a micro broth dilution method. Clinical characteristics and drug-resistance rates were retrospectively reviewed, and the predictors of mortality were evaluated by univariate and multivariate analyses.

**Results:**

The mortality rate of AP patients complicated with MDR-*K. pneumoniae* infections reached 46.5% (33 of 71), and pancreas (n = 53) was the most common site of MDR-*K pneumoniae* strains. The drug resistance rates of MDR-*K. pneumoniae* were high to 11 of 12 common antibiotics (more than 50.0%) except of tigecycline (23.9%). The predictor independently associated with mortality was septic shock (hazard ratio 2.959, 95% confidence intervals 1.396 – 6.272, *P* = 0.005).

**Conclusions:**

More attention should be paid for pancreatic MDR-*K. pneumoniae* infections among AP patients The predictor for mortality of AP patients complicated with MDR-*K. pneumoniae* infection is septic shock. Therefore, further clinical investigations should focus on areas against septic shock.

## Introduction

Acute pancreatitis (AP) has been a commonly acute abdominal disease with increasing incidence and mortality rate during the past decade [1]. Infectious complications often develop in the middle-late stages, which are the major causes for second death peak of AP. The mortality rate for AP, especially severe AP (SAP) patients, complicated with bacterial infections was up to 70% [2–4]. Besides, there were 30% of AP patients subsequently suffered from (peri)pancreatic or extrapancreatic infections [3].

Due to prolonged hospitalizations and exposure to overused antibiotics, AP patients have become a high-risk population for multidrug-resistant (MDR) bacterial infections [5]. Recently, several studies have showed that a rising proportion of AP patients with MDR bacterial infections reached 63% [5–9]. MDR-*K. pneumoniae* has a strong drug-resistant ability through several mechanisms including target alteration, drug inactivation, decreased cell permeability, and increased efflux pump activity [10–13]. MDR-*Klebsiella pneumoniae* is found as a dangerous pathogen for hospital-acquired infections because of the limited antibiotic regimens [14–16]. Similarly, some studies have given an account of MDR bacterial infections among AP patients which aimed to differentiate between MDR and non-MDR bacterial infections, but devoted to the aspect of the drug-resistance and predictors for mortality of AP patients complicated with MDR infections [5, 15, 17]. In addition, the availability of regional drug-resistance profiles is likely to improve the appropriateness of anti-infective treatments, and the predictors for mortality of AP patients with MDR bacterial infections need to be identified in order to raise the survival rate.

Up to now, there only few case reports have paid attention to the MDR-*K. pneumoniae* infection in AP, which suggest that the knowledge related to MDR-*K. pneumoniae* infection among AP patients is still needed to be enhanced [7, 18]. The present bi-centric retrospective cohort study has devoted to investigate the drug-resistance and the predictors of overall mortality among AP patients complicated with MDR-*K. pneumoniae* infection, thereby making progress in the prognosis in this population.

## Methods

### Ethics committee approval

Due to the property of retrospective cohort study, our study would not interfere with the enrolled patients. Institutional Review Board of Xiangya Hospital (No. 202103047) and the Third Xiangya Hospital (No. 21019) approved the waiver of patient informed consent. This study and all methods have been performed in accordance with the Declaration of Helsinki and obtained ethics approval to collect data from Ethic Committee of the Xiangya Hospital of Central South University (No. 202103047) and IRB of The Third Xiangya Hospital of Central South University (No. 21019). Information was gathered from electrical medical system in an anonymous manner. The authors ensure patient data confidentiality.

### Study design and setting

Seventy-one AP patients who developed MDR-*K. pneumoniae* infections, in Xiangya Hospital, a 3500-bed tertiary-care teaching hospital, and Third Xiangya Hospital, an 1800-bed tertiary-care teaching hospital, Central South University, Changsha, China, from August 1st, 2016 to August 1st, 2020 were retrospectively enrolled. The follow-up period to observe the clinical outcome lasted for 90 days from the day of onset of MDR-*K. pneumoniae* infection which was defined as the date of first MDR-*K. pneumoniae* positive sample collection (not reported time point from microbiology laboratory). Clinical characteristics, from electronical medical system, were recorded within 24 h after the time of entry into the cohort. Clinical outcomes were divided into mortality and survival to identify the independent predictors.

### Patients

In our cohort, diagnosis and classification of AP were based on the criteria of revised Atlanta classification [19]. Criteria of etiology are listed as follows: (1) biliary: choledocholithiasis or cholelithiasis by enhanced computed tomography; (2) hypertriglyceridemia: triglyceride level > 1000 mg/dl; (3) alcoholic: drinking > 50 g/d for at least 1 year. The onset of MDR-*K. pneumoniae* infection (study entry) indicated to the date of first positive sample collection. In addition, doctors prescribed the empirical antibiotic treatment to ‘suspected’ infections before getting the further identification and drug-resistance for targeted therapy. The initial diagnoses of 71 patients were moderately SAP and SAP since most mild AP patients were treated in lower-level hospitals. There were 38 patients in Xiangya hospital and 33 patients in Third Xiangya hospital enrolled. We excluded patients < 16 or > 80 years old, with a history of chronic pancreatitis or pancreatic surgery, as well as with no positive specimen result.

### Treatment protocol of AP

According to the latest international guidelines Patients were assessed and managed by the multidiscipline team in the early stage of admission, including pancreatic surgeons, radiologists, intensive care unit and gastroenterology physicians. Conservative treatments were taken for (peri)pancreatic necrosis without evidence of secondary infections to intensely observe any changes in the conditions of the patients. Antibiotics (cefoperazone–sulbactam, carbapenems and so on) were previously prescribed when necessary and were adjusted according to the subsequent microbiology profile.

We collected specimens from the ‘suspected’ infected patients, including (peri)pancreatic necrosis, blood, bronchial alveolar lavage fluid as well as bile, then we immediately transported them to clinical microbiology laboratory for identifications of the causative pathogens. In both 2 tertiary medical centers, MDR-*K. pneumoniae* pancreatic infection was characterized as the (peri)pancreatic necrosis or fluid obtained with positive specimen from the first therapeutic intervention instead of the fine-needle aspiration. The therapeutic intervention was performed in AP patients, who are complicated with clinically confirmed (peri)pancreatic infection after failure of conservative treatment. The step-up approach included percutaneous catheter drainage or endoscopic transluminal drainage and if necessary, subsequent minimal access retroperitoneal necrosectomy, video-assisted retroperitoneal debridement, or endoscopic transluminal necrosectomy and open necrosectomy. In line with the latest international guidelines, treatment for infectious pancreatic necrosis was performed in accordance with patient’s condition according to [20].

### Definition

MDR-*K. pneumoniae* infections were defined with positive specimen results according to the criteria of the Centers for Disease Control [21]. On the basis of the Berlin Definition of acute respiratory distress syndrome, acute respiratory distress syndrome was diagnosed and classified [22]. Diagnostic methods of pancreatic infections included: (1) enhanced computed tomography showing the presence of extraluminal gas, and (2) positive specimen from pancreatic necrosis obtained by first drainage or necrosectomy. Septic shock was diagnosed in the patient who required vasoactive drugs for maintaining mean arterial blood pressure > 65 mmHg with value of serum lactate > 2 mmol/l after fluid resuscitation with MDR-*K. pneumoniae* infection [23].

### Microbiology

All specimens were collected by clinicians when patients were suspected for infections or first intervention for infected pancreatic necrosis (pancreatic infections), then we immediately transported specimens to microbiology laboratory in line with standard procedures [24]. Only the data at the first time of positive result was recorded for further statistical analysis when the MDR-*K. pneumoniae* pathogen occurred repeatedly in the same site of one patient. The specimen was injected into each bottle of a set of aerobic and anaerobic culture bottles and immediately transported to the clinical microbiology laboratory. Identification and antimicrobial susceptibility testing of MDR-*K. pneumoniae* pathogen were carried out using the Vitek-2 system (bioMe´rieux, Marcy L’etoile, France). Minimum inhibitory concentratio was measured via a micro broth dilution method following the standards of National Committee for Clinical Laboratory. Positive specimen and drug-resistance results were reported at the same time from microbiology laboratory [24]. Breakpoints of minimum inhibitory concentration (susceptible, resistant) for each antibiotics were according to the Clinical Laboratory Standard Institute guidelines: amoxicillin-clavulanate (≤ 8/4, ≥ 32/16 μg/ml), amikacin (≤ 16, ≥ 32 μg/ml), aztreonam (≤ 4, ≥ 16 μg/ml), ciprofloxacin (≤ 0.25, ≥ 1 μg/ml), gentamicin (≤ 4, ≥ 16 μg/ml), imipenem (≤ 2, ≥ 8 μg/ml), meropenem (≤ 2, ≥ 8 μg/ml), levofloxacin (≤ 0.5, ≥ 2 μg/ml), sulfamethoxazole (≤ 2/38, ≥ 4/76 μg/ml), piperacillin-tazobactam (≤ 16/4, ≥ 128/4 μg/ml), ceftriaxone (≤ 8, ≥ 64 μg/ml) and tigecycline (≤ 2, ≥ 8 μg/ml). MDR-*K. pneumoniae* was defined as the strain non-susceptible to at least one agent in three or more antimicrobial categories [10].

### Statistical analysis

For continuous variables, data were expressed as mean (± SD) and compared with Student’s t-test or Mann–Whitney-U test. Categorical variables were compared with the *χ*^2^ test or Fisher exact tests. Only the characteristics with value of *P* < 0.05 can enter the multivariate analysis. In multivariate analysis, survival time was put into Cox regression analysis to confirm the independent predictors associated with overall mortality. Hazard ratio and 95% confidence interval were calculated to estimate the degree of proportional hazard. Survival distribution for the independent predictors was described via Kaplan–Meier curve. A value of *P* < 0.05 (two-tailed) was defined as statistically significant, and SPSS 24.0 (IBM SPSS Statistics, IBM Corp., Armonk, NY, US) was used for all statistical analysis.

## Results

**Fig. 1:**
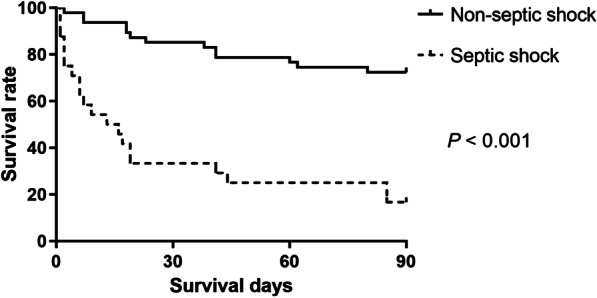
Kaplan–Meier curve estimated survival rates between septic shock and non-septic shock group (16.7% vs. 72.3%, *P* < 0.001)

During the 4-year-study period, 71 AP patients experienced MDR-*K. pneumoniae* infections. The etiology of 71 patients was categorized as biliary (n = 30, 42.3%), hyperlipidemic (n = 26, 36.6%), alcoholic (n = 3, 4.2%) and other causes (n = 12, 16.9%). The mean age of the patients was 48.8 years. 56 (78.9%) and 15 (21.1%) patients were male and female, respectively. Forty-three patients (60.6%) were classified as SAP, and 28 patients (39.4%) were moderately SAP. Twenty-four (33.8%) patients met criteria for septic shock, and 27 (38.0%) patients required invasive mechanical ventilation support. Sixty-three patients (88.7%) were referred from other hospitals 2 days after onset of AP with a median referral time of 19.8 days. Fifty-seven patients (80.3%) occurred multiple organisms or sites infections. *Acinetobacter baumannii* infections (n = 33, 46.5%) were most common co-infections, followed by *Klebsiella ozaenae* (n = 14, 19.7%), *Pseudomonas aeruginosa* (n = 11, 15.5%) and *Burkholderia cepacia* (n = 11, 15.5%). The mortality of AP patients complicated with MDR-*K. pneumoniae* infection was 46.5% (33 of 71). Percutaneous catheter drainage to minimal access retroperitoneal necrosectomy was most commonly therapeutic intervention (n = 19) for pancreatic infections (only 53 patients), follow by step-up to open necrosectomy (n = 10), only percutaneous catheter drainage (n = 7) or endoscopic transluminal drainage (n = 7), open necrosectomy (n = 4), percutaneous catheter drainage to video-assisted retroperitoneal debridement (n = 3) and endoscopic transluminal drainage to endoscopic transluminal necrosectomy (n = 3). The details of clinical characteristics and comparison between alive and death group are presented in Table 1. In the univariate analysis, multiple infected organisms or sites (*P* = 0.042), SAP (*P* = 0.003), invasive mechanical ventilation (*P* < 0.001), sepsis shock (*P* < 0.001) and hemorrhage (*P* < 0.001) were significantly related to mortality. In the multivariable analysis (Table 2), only septic shock (hazard ratio 2.959, 95% confidence interval 1.396—6.272, *P* = 0.005) was the independent predictor of overall mortality. In Fig. 1, compared with patients without septic shock, the survival rate was significantly lower in the patients suffered from septic shock (16.7% vs. 72.3%, *P* < 0.001).

**Table 1:** Clinical characteristics and comparison between survival and mortality of 71 AP patients with MDR-*K. pneumoniae* infections

Characteristics	Total	Survival (n = 38)	Mortality (n = 33)	*P*
Age, years (mean ± SD)	48.8 ± 12.7	49.5 ± 11.8	48.1 ± 13.6	0.632
Sex, n (%)				0.571
Male	56 (78.9)	29 (76.3)	27 (81.8)	
Female	15 (21.1)	9 (23.7)	6 (18.2)	
Etiology, n (%)				0.688
Biliary	30 (42.3)	15 (39.5)	15 (45.5)	
Hypertriglyceridemia	26 (36.6)	14 (36.8)	12 (36.4)	
Alcohol	3 (4.2)	1 (2.6)	2 (6.1)	
Others	12 (16.9)	8 (21.1)	4 (12.1)	
Classification of AP, n (%)				0.003*
Moderately severe AP	28 (39.4)	21 (55.3)	7 (21.2)	
Severe AP	43 (60.6)	17 (44.7)	26 (78.8)	
Primary site of infections, n (%)				0.744
Pancreas (peri)	53 (74.6)	29 (76.3)	24 (72.7)	
Bloodstream	9 (12.7)	4 (10.5)	5 (15.2)	
Lung	8 (11.3)	4 (10.5)	4 (12.1)	
Biliary tract	1 (1.4)	1 (2.6)	0	
Multiple infected organisms or sites, n (%)	57 (80.3)	27 (71.1)	30 (90.9)	0.042*
Concomitant infected bacteria				
*Acinetobacter baumannii*	33 (46.5)	16 (42.1)	17 (51.5)	0.428
*Klebsiella ozaenae*	14 (19.7)	5 (13.2)	9 (27.3)	0.136
*Pseudomonas aeruginosa*	11 (15.5)	7 (18.4)	4 (12.1)	0.464
*Burkholderia cepacia*	11 (15.5)	4 (10.5)	7 (21.2)	0.215
*Escherichia coli*	8 (11.3)	6 (15.8)	2 (6.1)	0.359
*Proteus mirabilis*	5 (7.0)	4 (10.5)	1 (3.0)	0.363
*Enterobacter cloacae*	5 (7.0)	1 (2.6)	4 (12.1)	0.176
Referred patient, n (%)	63 (88.7)	34 (89.5)	29 (87.9)	1.000
Time of infection from onset of AP, n (%)				0.290
< 14 days	6 (8.5)	5 (13.2)	1 (3.0)	
15–30 days	29 (40.8)	14 (36.8)	15 (45.5)	
> 30 days	36 (50.7)	19 (50.0)	17 (51.5)	
Respiratory function, n (%)				0.811
Mild ARDS	61 (85.9)	33 (86.8)	28 (84.8)	
Severe and moderate ARDS	10 (14.1)	5 (13.2)	5 (15.2)	
Sepsis shock, n (%)	24 (33.8)	4 (10.5)	20 (60.6)	< 0.001*
Invasive mechanical ventilation, n (%)	27 (38.0)	7 (18.4)	20 (60.6)	< 0.001*
Antibiotic therapy, n (%)				0.386
Carbapenem (high dose, extended infusion)	25 (35.2)	14 (36.8)	11 (33.3)	
Tigecycline	6 (8.5)	3 (7.9)	3 (9.1)	
Penicillins/β-lactamase inhibitors	6 (8.5)	6 (15.8)	0	
Quinolone	4 (5.6)	3 (7.9)	1 (3.0)	
Carbapenem and tigecycline	21 (29.6)	6 (15.8)	15 (45.5)	
Carbapenem and penicillins/β-lactamase inhibitors	4 (5.6)	4 (10.5)	0	
Carbapenem and sulphonamides	3 (4.2)	1 (2.6)	2 (6.1)	
Polymyxins and fosfomycin	2 (2.8)	1 (2.6)	1 (3.0)	
Hospitalization, days (mean ± SD)	45.8 ± 28.8	41.2 ± 33.3	49.9 ± 24.1	0.214
Major complications, n (%)				
Hemorrhage	23 (32.4)	4 (10.5)	19 (57.6)	< 0.001*
I ntestinal leakage	15 (21.1)	5 (13.2)	10 (30.3)	0.078
Pancreatic fistula	8 (11.3)	5 (13.2)	3 (9.1)	0.716

*P values are statistically significant between survival and mortality group

*SD* standard deviation, *AP* acute pancreatitis, *ARDS* acute respiratory distress syndrome

**Table 2:** Multivariate analysis of predictors for mortality in AP patients complicated with MDR-*K. pneumoniae* infections

Variable	HR (95% CIs)	*P*
SAP	2.182 (0.866–5.502)	0.098
Male	0.677 (0.257–1.786)	0.431
Age > 50 years	1.335 (0.609–2.926)	0.471
Multiple infected organisms or sites	1.366 (0.394–4.742)	0.623
Hemorrhage	1.315 (0.607–2.847)	0.487
Invasive mechanical ventilation	1.961 (0.896–4.295)	0.092
Septic shock	2.959 (1.396–6.272)	0.005

*HR* hazard ratio, *CI* confidence interval

Drug-resistance rate and distribution of MDR-*K pneumoniae* strains are shown in Table 3. Pancreas(peri) (n = 53) was the most common site of MDR-*K pneumoniae* pathogens in AP patients, followed by bloodstream (n = 32), lung (n = 27) and biliary tract (n = 1). Drug-resistance rates of MDR-*K pneumoniae* to 11 of 12 antibiotics were more than 50%, including amoxicillin-clavulanate, amikacin, aztreonam, ciprofloxacin, gentamicin, imipenem, meropenem, levofloxacin, sulfamethoxazole, piperacillin-tazobactam, ceftriaxone, except tigecycline (23.9%).

**Table 3:** Resistance rates of 113 MDR-*K.pneumoniae* strains to 12 antibiotics according to the different sites of infections

Antimicrobial	Pancreas (peri) (n = 53)	Bloodstream (n = 32)	Lung (n = 27)	Biliary tract (n = 1)	Total strains (n = 113)
Amoxicillin-clavulanate	53 (100)	32 (100)	26 (96.3)	1 (100)	112 (99)
Amikacin	28 (52.8)	18 (56.2)	17 (63)	0 (0)	63 (55.8)
Aztreonam	45 (84.9)	29 (90.6)	26 (96.3)	1 (100)	101 (89.4)
Ciprofloxacin	44 (83)	29 (90.6)	24 (88.9)	1 (100)	98 (86.7)
Gentamicin	39 (73.6)	26 (81.3)	25 (92.6)	0 (0)	90 (79.6)
Imipenem	38 (71.7)	26 (81.3)	24 (88.9)	1 (100)	89 (78.8)
Meropenem	38 (71.7)	26 (81.3)	23 (85.2)	1 (100)	88 (77.9)
Levofloxacin	42 (79.2)	29 (90.6)	25 (92.6)	1 (100)	97 (85.8)
Sulfamethoxazole	20 (37.7)	14 (43.8)	11 (40.7)	1 (100)	67 (59.3)
Piperacillin-tazobactam	41 (77.4)	29 (90.6)	24 (88.9)	1 (100)	95 (84.1)
Ceftriaxone	46 (86.8)	30 (93.8)	27 (100)	1 (100)	104 (92.0)
Tigecycline	15 (28.3)	7 (21.9)	5 (18.5)	0 (0)	27 (23.9)

Values are no. (%) of resistant strains, except as indicated

## Discussion

In the past decade, the rapidly emerging MDR pathogens associated infections with limited therapeutic options has become a global threat to medical systems [11, 17]. The crisis of MDR bacterial infection was more frequently occurring in developing countries. *K. pneumoniae*, as the second most common nosocomial gram-negative bacterium, leads to significantly high all-cause mortality in worldwide [10, 12, 14, 25]. Carbapenem, amikacin and tigecycline used to be effective and latest antibiotics for MDR-*K. pneumoniae* treatments, but recent researches revealed that drug-resistance rates of these three antibiotics were significantly increasing which may be associated with overuse during treatments [24, 26]. In this era of MDR bacterial infections, clinician in all departments should pay more attentions on the preventions and treatments of MDR-*K. pneumoniae* infections [13, 17].

Infection is one of the most common complications in AP patients due to prolonged hospitalization and excessive anti-inflammatory therapy [1, 6, 9, 27]. To our knowledge, this is the largest retrospective-cohort-study investigating the predictors of mortality in AP patients complicated with MDR-*K. pneumoniae* infections. It is consistent with previous studies that biliary etiology was the most common etiology for MDR-*K. pneumoniae* infections [5, 28]. In line with Ning et al., our study found that there was a higher mortality (46.5%) of AP patients complicated with MDR-*K. pneumoniae* infections which confirmed that MDR-*K. pneumoniae* has played a lethal role in the procedure of AP patients [5, 15].

The main finding of our cohort study is that the independent predictor of mortality among AP patients with MDR-*K. pneumoniae* infections was septic shock. Immune suppression in early stage of AP may result in excessive systemic inflammatory response syndrome, inducing intestinal barrier dysfunction, thereby increasing intestinal mucosal permeability. Infections of AP patients may be induced by enteric pathogens that translocated into the parenteral system, especially bloodstream, to cause infection due to intestinal barrier disruption [29]. Septic shock is the most severe infectious complication in bloodstream infections. Early recognition and successful management of septic shock remain numerous knowledge gaps and challenges, because SAP patients without infections may mimic septic shock [30]. Patients with septic shock should be timely received antibiotic treatments, because there is a definite guideline to start anti-sepsis shock therapy as early as possible [31]. It is the first report described hazards of septic shock for AP patients complicated MDR-*K. pneumoniae* infections, which alerts clinicians to prevent barrier disfunction as well as bacterial translocation so as to prompt timely diagnosis and treatment.

Jain et al. revealed that infected pancreatic necrosis infected by MDR bacteria rather than non-MDR bacteria, was an independent predictor of mortality in AP patients, however, it was not significantly related to death in our study [9]. The possible reason might be all our enrolled patients were suffered from MDR bacterial infections with considerable proportion of multiple sites which may reduce the hazard of mono-pancreatic infections in the univariate analysis. The analysis of the intervention for infectious pancreatic necrosis was not the focus of our study which would be done in future.

Consistent with the previous analysis of infections, our microbiologic result demonstrated that bloodstream (n = 32) was the most common extra-pancreatic site of MDR-*K. pneumoniae* infections which may verify our hypothesis for bacterial translocation to the bloodstream [32]. According to previous studies, drug resistance of MDR-*K. pneumoniae* pathogens causing AP secondary infections was quite serious [5, 15]. In our cohort, most strains were carbapenem-resistant *K. pneumoniae*, which may be associated with prophylactic antibiotic treatment and high referral rate [27]. Li et al. reported that carbapenem was considered as the front-line regimen for AP patients with ‘suspected’ pancreatic infections [17]. By contrast, Tugal et al. oppositely reported that imipenem/cilastatin was failed to treat a carbapenem-resistant *K. pneumoniae* infected necrotizing pancreatitis[7]. Concerning this conflicting issue, our results suggested that carbapenems (regular dose) may not be effective antibiotics against MDR-*K. pneumoniae* due to the high drug-resistance rate and augmented renal clearance [33, 34]. Prophylactic use of antibiotics, especially carbapenems, may not be recommended in AP for the prevention of infectious complication [20]. MDR-*K. pneumoniae* has become a growing threat, and it is necessary for clinicians to enhance the administration of antibiotics. However, the phenomenon of overuse and misuse of antibiotics for AP, perhaps resulting in drug-resistant bacteria, has soared worldwide, especially in China [35].

Similar as Moka et al. reported, our cohort showed that tigecycline is recommended as one of last-line antibiotics, whereas carbapenem may be substituted by tigecycline for MDR-*K. pneumoniae* infection treatment [15]. However, it was reported that tigecycline was overuse with risk of increasing drug-resistance rate in China [24]. Though polymyxin played an essential role in the antibiotic treatment for MDR or extensively drug-resistant bacterial infections, polymyxin resistance assays were lacked for most of patients in our study [36]. Ceftazidime avibactam, reported with better effects than tigecycline, has been approved, by Chinese Food and Drug Administration, to treat with MDR-*K. pneumoniae* infections during recent years [37]. Prescribing combined or novel antibiotic therapy to all the MDR-*K. pneumoniae* infections, including extensively-drug-resistant or pan-drug-resistant *K. pneumoniae*, is not a reasonable therapeutic option due to the risk of increasing resistance. In this era of MDR, further steps are advised to facilitate molecular or pharmacologic research to potentially accelerate the process of finding novel antimicrobial breakpoints and clearly reveal the mechanism of drug resistance. Furthermore, management of antibiotics would affect drug-resistances of pathogens. However, it may be too difficult to analyze the data on the antibiotic therapy into several subgroups because of limited number of patients which need to be revealed with standardized regimens in the prospective studies.

This double-center retrospective cohort study has several limitations. Firstly, due to its retrospective nature, our study was limited by nonincluded factors, insufficient information as well as selection biases. As the patients in two large tertiary hospital in Central China, most of patients were referred from other hospitals without precise details of antibiotic exposures before referral which may have impact on the outcomes and need to be verified in prospective multi-center studies. Secondly, in the era of highly advanced medical technologies, the novel molecular technology such as third generation sequencing might be used to promote rapid diagnoses and reveal the mechanism of MDR-*K. pneumoniae* infections. Thirdly, it’s noted that the comparison of intervention for infected pancreatic necrosis was lacked, because some patients were only diagnosed with proven extrapancreatic infections. What’s more, our analysis only investigated the predictors of overall mortality instead of infection-related mortality as a result of limited number with no ability for excluding the death of polymicrobial infections or major complications which were included as potential predictors. Finally, our findings should be carefully interpreted in the lowly drug-resistant region. Notably, the 4-year-period in two large hospitals in China is the huge advantage of our cohort.

## Conclusions

MDR-*K. pneumoniae* infection is recognized as a serious complication with high mortality among AP patients. MDR-*K. pneumoniae* pathogens, most frequently found in (peri)pancreas, are highly resistant to common antibiotics except for tigecycline. We firstly reveal that the independent predictor for the mortality of AP patients complicated with is septic shock. Further clinical investigations should focus on areas against septic shock.

## Data Availability

The datasets used and/or analyzed during the current study are available from the corresponding author on reasonable request.

## Abbreviations

AP: Acute pancreatitis
SAP: Severe acute pancreatitis
MDR: Multidrug-resistant
